# Aging‐related changes of cognitive performance and stress regulation in high functioning elderly individuals

**DOI:** 10.1111/sms.13374

**Published:** 2019-04-29

**Authors:** Thomas Finkenzeller, Sabine Würth, Erich Müller, Günter Amesberger

**Affiliations:** ^1^ Department of Sport Science and Kinesiology University of Salzburg Hallein Austria

**Keywords:** cognition, habituation, longitudinal study, mental stress, practice effect, repetition of measurements, septuagenarians

## Abstract

This article aims to analyse long‐term changes in cognitive performance and psychophysiological stress regulation in a specific sample of 10 young‐old (age at pre‐test: M ± SD = 63.2 ± 1.5) and 12 old‐old (age at pre‐test: M ± SD = 69 ± 2) persons possessing a high psychosocial status and a physically active lifestyle. Psychophysiological parameters were recorded prior to, during, and after the administration of a cognitive performance test battery. The measurements took place three times within a 6‐month period, and again 6 years later in a follow‐up test. Considering practice and habituation effects, findings provide no compelling evidence of an aging‐associated cognitive decline in attention, multiple choice reaction performance, and information processing speed, either in the young‐olds, or in the old‐olds. Furthermore, psychophysiological stress regulation showed no long‐term alteration regarding skin conductance level and heart rate. Based on these findings, it is assumed that psychosocial health and physical activity might contribute to the preservation of cognitive abilities and stress regulation into the 70s. Finally, this study demonstrated the significance of considering practice and habituation effects elicited through test repetitions in order to estimate long‐term effects.

## 1 INTRODUCTION

Aging is characterized by changes of psychological and biological factors across one's life span.^1^ There is evidence that a decline in cognitive performance occurs around 60 years of age.^2^, ^3^, ^4^, ^5^ However, it was shown that the cognitive decline is small in the elderly with good functioning in baseline.^6^ Alterations in cognitive function appear to accompany changes in physiological stress regulation.^7^, ^8^ The maintenance of brain health^9^ and stress regulation^10^ has a significant impact on the ability to function adequately and independently in everyday life.^1^ There is promising evidence that engagement in intellectual, physical, and social activities may contribute to a more gradual decline in cognitive performance.^11^, ^12^ Aerobic exercise training appears to be beneficial on psychophysiological stress regulation.^13^, ^14^

According to Hertzog and colleagues,^15^ an individual's cognitive performance can fluctuate differently within life course after maturation. The high degree of freedom of how to live a life may contribute to variability in cognitive functions that increases with advanced age.^1^, ^11^ Nutrition and physical exercise influence cognitive enrichment directly through metabolic and physiological changes on the neural substrate.^15^, ^16^ A socially supportive environment,^17^ life satisfaction,^18^ and leading a cognitively demanding life^19^ are further enriching factors of decreased aging‐related cognitive loss. In this context, the question arises whether elderly individuals who exhibit a life including numerous health‐protective factors are able to postpone aging‐related cognitive decline beyond 70 years of age.

A methodological problem in longitudinal research on aging‐associated cognition is that the repetition of a cognitive task can mask aging‐related changes, resulting in an underestimation of such changes.^3^ Depending on the complexity of the task and age of the person, practice effects in specific tasks of intelligence tests can persist as long as 7 years.^20^ In tasks measuring aspects of memory, speed, and spatial visualization, it was shown that practice effects are still observable approximately 2.5 years.^21^ So far, no clear solution has been formulated to overcome this problem. This study considered retest effects through three measurements within a period of 6 months to which findings of the follow‐up test were related.

Previous studies have shown that increasing levels of perceived acute and chronic stress are associated with accelerated declines in cognitive function, in particular among adults aged 60 years and older.^10^, ^22^, ^23^ However, there is little knowledge about changes in psychophysiological stress parameters over time and the relationship with cognitive performance. Monitoring psychophysiological activity during the anticipation of, administration of, and recovery from psychologically stressful tasks^24^ provides information on one's ability to cope with cognitively demanding tasks. With advancing age, the impact of sympathetic activity increases,^25^ whereas a mitigation of parasympathetic activity is observed.^26^ Consequently, an aging‐related increase of sympathetic activity might occur in stressful situations, reflecting a limited psychophysiological coping ability. Accordingly, skin conductance level (SCL) as a parameter of sympathetic activity,^27^ heart rate (HR) as an index of sympathovagal balance,^28^ and respiration rate (RR) as a control variable^29^ were used in this study. Similar to practice effects in cognitive tasks, the repeated administration to cognitive stressors elicits an alleviated psychophysiological activity over time as participants become familiar with the test setting.^24^ The above mentioned specific longitudinal design attempts to control for habituation effects.

Data reported in this article resulted from a multidimensional intervention study on alpine skiing, indicating no impact of the intervention on cognition and psychophysiological reactivity and recovery.^30^ Hence, the question on sustainability of intervention effects does not arise. Therefore, data of the Salzburg Skiing for the Elderly Study (SASES) were analyzed focusing on long‐term changes in cognitive performance and stress regulation in two age groups of elderly persons, in young‐olds (<66 years) and old‐olds (≥66 years), considering the impact of two repetitions of a mental stress test battery in a short‐term interval of half a year. A sample of successfully aging elderly individuals exhibiting a high level of health‐promoting resources in terms of behavioral and psychosocial variables (see first article within this supplement) were hypothesized (a) to postpone age‐associated cognitive decline into the 70s and (b) to demonstrate no acute changes in psychophysiological regulation during the cognitive tasks regarding a period of nearly 6 years. Practice and habituation effects are taken into account due to a specific longitudinal design consisting of three measurements in a close interval of 6 months and again in a follow‐up testing 6 years later, indicating possible aging‐related alterations.

## 2 METHODS

### 2.1 Subjects

The sample is characterized in detail regarding the educational status, psychological well‐being and psychosocial variables including life satisfaction, self‐concept, health status, and self‐efficacy in the first article of this supplement. A Mann‐Whitney test indicated that there was no significant difference in the educational status among the age groups, *U* = 52, *P* = 0.57. According to their ages, 7 females and 3 males (*M*age = 63.2 ± 1.5 years at t1) were assigned to a young‐old (<66 years), and 6 females and 6 males (*M*age = 69.0 ± 2.0 years at t1) to an old‐old group (≥66 years), which comes close to the classification of Xu, Qiu, Gatz, Pedersen, Johansson, Fratiglioni.^31^ Thirteen subjects took drugs regularly for medical reasons. However, only six subjects suffered from one or more chronic diseases such as coronary heart disease (n = 1 young‐old and 1 old‐old), diabetes mellitus (n = 2 young‐olds), hypertension (n = 1 old‐old), sleeping disorders (n = 1 young‐old), prostate cancer (n = 1 old‐old), or hypercholesterolemia (n* *= 1 young‐old). A number of 4‐6 individuals (depending on the parameter) were excluded from analyses of RR, HR, and SCL due to artifacts. The respective sample sizes for psychophysiological parameters, as well as for objective performance are mentioned in the results.

### 2.2 Study design

A longitudinal design with four measurements of different intervals between tests was applied. The first three tests were conducted in the context of the SASES (for details see the article on study design in this supplement) showing no effect of an alpine skiing intervention on cognitive performance and psychophysiological regulation. Thus, data of SASES appeared to be appropriate to examine whether aging in two age groups results in alterations of cognitive performance and psychophysiological stress regulation regarding a time span of nearly 6 years. This design has the advantage that long‐term changes can be interpreted under the perspective of short‐term practice and habituation effects.

### 2.3 Test protocol

Upon arrival at the laboratory, participants filled out a questionnaire on motivational well‐being. Afterward, electrocardiogram (ECG) electrodes were attached in accordance to lead II chest placement.^32^ Electrodes for recording of SCL were placed on the medial phalanx of the forefinger and the ring finger of the non‐dominant hand, and a respiration sensor was attached at the level of the umbilicus.

The test started with a naive relaxation (NR) phase, in that participants had were asked to relax with closed eyes in an armchair and were asked not to think about anything in particular for five minutes. This procedure was the same for the adjacent paced relaxation (PR1) and the paced relaxation phase at the end of the stress test (PR2). In PR1 and PR2, respiration rate (RR) per minute was two cycles lower than in the NR. The RR was acoustically presented by simulating inhaling and exhaling. The presentation of a lowered and standardized breathing frequency encouraged clients to relax and build up a high heart rate variability (HRV).^33^ In the time interval between the paced relaxation phases, three cognitive performance tests were administered to induce mental stress. The mental stress phase started with a general attention test (cognitrone) that followed a multiple choice reaction performance under time pressure (determination test) and ended with an information processing speed test (Zahlenverbindungstest).

### 2.4 Measures

Motivational well‐being was recorded by the PANAVA,^34^ which consists of 10 items and assesses the three dimensions of positive activation, negative activation, and valence. The items are answered using a bipolar scale with seven steps.

Cognitive performance was evaluated by administering two tests of the Vienna Test System (Schuhfried^®^, Mödling, Austria), and an information processing task. According to the test manuals, the proposed aiming variables were extracted for further analyses.

General attention ability was assessed by the cognitrone (COG; version S8) task.^35^ In this test, four geometrical figures have to be compared with a further geometrical figure that is presented above the four geometrical figures. The task is to indicate if one geometrical figure out of the four is congruent to the figure above by pressing the corresponding button. The presentation time of the stimuli is adapted to the individual working speed. The test duration is seven minutes. Dependent variables chosen were the sum of all reactions reflecting information processing speed and error rate in percent providing information accuracy.

Multiple choice reaction performance under time pressure was measured with the determination test (DT; version S1; adaptive mode).^36^ The participants had to react on color stimuli, direction stimuli, and acoustic stimuli as fast and precise as possible by pressing corresponding buttons on a response panel and a foot pedal. In the adaptive mode, the presentation speed is adjusted to the performance level. Hence, a subject processes the stimuli at the individual reaction time threshold, provoking additional stress. The test takes 4 minutes to complete. Performance in DT is displayed by the total sum of correct reactions.

Finally, an information processing speed test, the Zahlenverbindungstest^37^ (ZVT, Number Connection Test), was filled out on paper with a pencil. Information processing time is reflected by the average of all four administered ZVT versions. Table 1 shows the intercorrelations among the test scores including the data of all four assessments.

**Table 1:** Intercorrelations (Pearson) among tests

Measure	n	1	2	3
1. COG sum reactions		‐		
2. COG errors (%)	87	0.13	‐	
3. DT correct reactions	87	0.44^a^	−0.06	‐
4. ZVT processing time	83	−0.48^a^	0.08	−0.41^a^

*Note*.

a *P* < 0.001.

Physiological data were recorded using a NeXus‐10 device and software Biotrace+ (mindmedia©, Roermond‐Herten, the Netherlands). The sample rate was set to 2048 Hz for the ECG and to 32 Hz for SCL and RR. Data processing of HR, RR, and SCL were conducted by using the software Biotrace+ for NeXus‐10 (version 2012C; mindmedia©). HR and respiration rate were analyzed for the last 4 minutes of PR1, COG, and PR2. The mean of the last 30 s of PR 1, COG, and PR2 were selected for analyses of SCL.

### 2.5 Statistics

All statistical analyses were performed with the software IBM SPSS Statistics for Windows (Version 24.0; IBM Corp., Armonk, NY, USA). The data are presented as mean (M) and standard deviation (SD). Results of the Shapiro‐Wilk test revealed that most of the variables demonstrated a normal distribution. The few non‐normal distributed variables marginally violated the assumption of normality. Thus, only parametric tests were used in accordance to Nimon.^38^ Alterations in motivational well‐being were analyzed by using a 4 × 2 (Time [t1, t2, t3, t4] × Age Group [young‐old, old‐old]) multivariate analyses of variances with repeated measures (MANOVA‐RM). Subsequently, univariate tests were conducted. Cognitive performance data were examined by running 4 × 2 (Time [t1, t2, t3, t4] × Age Group [young‐old, old‐old]) analyses of variances with repeated measures (ANOVAs‐RM). Regarding changes of RR, HR, and SCL, 4 × 3 × 2 (Time [t1, t2, t3, t4] x Block [PR1, COG, PR2] × Age Group [young‐old, old‐old]) ANOVAs‐RM were calculated. Greenhouse‐Geisser values are reported in the case of the violation of sphericity. When a significant time effect occurred, analyses were followed by a least significant difference (LSD) post hoc test, and the respective Cohen's *d* effect sizes are reported. Significant post hoc findings are displayed for effects that concern exclusive differences between t4 and the three measurements that were conducted more than 5 years prior to t4 in order to reflect the effect of repeating a measurement on the estimation of long‐term effects. Significance level was set at *P* < 0.05.

## 3 RESULTS

### 3.1 Changes of motivational well‐being prior to stress testing

Table 2 displays descriptive statistics on motivational well‐being prior to measurements.

**Table 2:** Descriptive statistics (mean ± standard deviation) of the PANAVA questionnaire (scale from 1 to 7)

Measure	*n*1/*n*2	December 2008/January 2009		April 2009		June 2009		April 2015	
		Young‐old	Old‐old	Young‐old	Old‐old	Young‐old	Old‐old	Young‐old	Old‐old
Positive activation	10/12	5.5 ± 0.6	5.8 ± 0.8	5.1 ± 0.9	5.69 ± 1.0	5.2 ± 1.0	5.5 ± 1.2	4.7 ± 0.6	5.0 ± 0.9
Negative activation	10/12	2.6 ± 1.2	1.9 ± 0.9	2.6 ± 0.9	2.04 ± 1.3	2.5 ± 0.9	1.9 ± 0.7	3.1 ± 0.7	2.7 ± 1.2
Valence	10/12	6.1 ± 0.8	6.0 ± 1.0	5.6 ± 1.0	5.46 ± 1.5	5.7 ± 0.9	6.0 ± 0.8	5.5 ± 0.8	5.5 ± 0.9

*Note.*

*n*1, sample size of age group young‐old; *n*2, sample size of age group old‐old.

MANOVA‐RM on well‐being revealed no significant effect of Age Group, [*F*(3, 18) = 1.95, *P* = 0.16, η² = 0.25, 1*‐*β = 0.42], and no interaction of Age Group × Time [*F*(9, 180) = 0.33, *P* = 0.96, η² = 0.02, 1*‐*β = 0.17]. However, a significant effect of Time was observed, [*F*(9, 180) = 2.06, *P* = 0.036, η² = 0.09, 1*‐*β = 0.86]. Subsequent univariate tests demonstrated a significant time effect for positive activation, [*F*(2.54, 36.43) = 4.07, *P* = 0.016, η² = 0.17, 1*‐*β = 0.77]. LSD comparison yielded significantly lower positive activation in the follow‐up test compared to t3 (*P* = 0.035, *d *= 0.50), t2 (*P* = 0.028, *d* = 0.56), and t1 (*P* < 0.001, *d* = 1.13). Univariate tests on negative activation and valence yielded no significant time effect.

### 3.2 Changes of cognitive performance

Descriptive data on cognitive performance are presented in Table 3.

**Table 3:** Descriptive statistics (mean ± standard deviation) of performance in COG (general attention ability), DT (multiple choice reaction performance under time pressure), and ZVT (information processing speed)

Measure	*n*1/*n*2	December 2008/January 2009		April 2009		June 2009		April 2015	
		Young‐old	Old‐old	Young‐old	Old‐old	Young‐old	Old‐old	Young‐old	Old‐old
COG sum reactions	9/12	327.1 ± 50.2	285.2 ± 45.7	341.6 ± 47.2	307.6 ± 46.8	362.9 ± 34.8	331.2 ± 42.1	352.4 ± 55.8	336.0 ± 75.3
COG errors (%)	9/12	3.9 ± 5.8	1.4 ± 1.5	2.0 ± 1.6	0.8 ± 0.8	0.8 ± 0.7	1.4 ± 1.0	1.5 ± 1.3	1.8 ± 1.2
DT correct reactions	9/12	201.6 ± 27.9	202.3 ± 28.1	218.3 ± 20.9	215.5 ± 23.6	229.4 ± 20.2	218.7 ± 31.3	208.0 ± 38.7	206.7 ± 29.2
ZVT processing time (sec)	9/11	92.6 ± 17.1	109.6 ± 35.4	82.0 ± 17.0	87.6 ± 17.9	74.4 ± 11.7	81.4 ± 16.8	88.4 ± 21.4	105.8 ± 24.8

*Note.*

*n*1, sample size of age group young‐old; *n*2, sample size of age group old‐old.

A significant time effect was observed in the sum of reactions of the COG (general attention ability test), the sum of correct reactions of the DT (multiple choice reaction performance under time pressure), and processing time of the ZVT (information processing speed). The results are provided in Table 4. No group effects and no interaction effect of Time x Group were obtained. LSD comparison of the sum of correct reaction in the COG demonstrated significant differences between t4 and t2 (*P* = 0.038, *d *= 0.51), as well as t4 and t1 (*P* = 0.002, *d* = 0.86). The number of correct reactions in the DT was significantly reduced at t4 compared to t3 (LSD *P* = 0.002, *d* = 0.75). The processing time of the ZVT at t4 was significantly longer compared to t3 (LSD *P* < 0.001, *d* = 1.16) and t2 (LSD *P* = 0.005, *d* = 0.73).

**Table 4:** Results of Time × Group mixed‐factors ANOVAs of performance (general attention ability), DT (multiple choice reaction performance under time pressure), and ZVT (information processing speed)

Measure	Time					Group					Time × Group				
	*df*	*F*	*P*	η²	*1‐*β	*df*	*F*	*P*	η²	*1‐*β	*df*	*F*	*P*	η²	*1‐*β
COG sum reactions	1.68, 31.87	11.95	<0.001^a^	0.39	0.98	1, 19	2.27	0.15	0.11	0.30	1.68, 31.87	0.93	0.39	0.05	0.19
COG errors (%)	1.19, 22.64	2.36	0.14	0.11	0.34	1, 19	1.46	0.24	0.07	0.21	1.19, 22.64	2.54	0.12	0.12	0.36
DT correct reactions	3, 57	8.79	<0.001^a^	0.32	0.98	1, 19	0.11	0.75	0.01	0.06	3, 57	0.58	0.60	0.03	0.15
ZVT processing time	2.21, 39.76	13.52	<0.001^a^	0.43	0.99	1, 18	1.99	0.18	0.10	0.27	2.21, 39.76	1.17	0.32	0.06	0.25

*Note*.

a *P* < 0.001.

### 3.3 Changes of psychophysiological data

Table 5 displays psychophysiological data for PR1, COG, and PR2.

**Table 5:** Descriptive statistics (mean** ± **standard deviation) of RR, HR, and SCL

Measure	*n*1/*n*2		December 2008/January 2009		April 2009		June 2009		April 2015	
			Young‐old	Old‐old	Young‐old	Old‐old	Young‐old	Old‐old	Young‐old	Old‐old
RR	8/10	PR	13.4 ± 4.5	13.7 ± 7.0	15.2 ± 5.4	14.9 ± 5.0	13.6 ± 3.7	13.6 ± 4.6	14.9 ± 5.9	14.7 ± 4.7
		COG	21.9 ± 3.5	23.3 ± 3.7	22.6 ± 2.6	22.9 ± 2.7	22.1 ± 1.9	23.9 ± 2.7	22.4 ± 4.8	24.9 ± 2.8
		PR	11.3 ± 2.4	10.5 ± 3.1	12.6 ± 2.7	12.5 ± 3.0	12.7 ± 2.1	12.6 ± 3.1	14.0 ± 3.1	15.4 ± 5.5
HR	8/10	PR	72.5 ± 8.2	70.3 ± 14.5	69.3 ± 9.8	66.0 ± 11.6	72.0 ± 8.3	66.6 ± 12.5	74.8 ± 15.0	78.2 ± 12.9
		COG	81.2 ± 9.7	75.9 ± 8.5	75.3 ± 9.9	72.4 ± 10.2	78.0 ± 7.7	74.5 ± 14.4	79.6 ± 13.9	79.4 ± 13.8
		PR	68.8 ± 9.2	71.0 ± 13.9	68.4 ± 8.7	68.5 ± 10.7	68.3 ± 7.2	67.5 ± 13.7	73.6 ± 14.6	76.8 ± 13.7
SCL	8/8	PR	1.4 ± 0.6	1.7 ± 1.3	2.0 ± 2.8	2.1 ± 2.0	1.2 ± 0.7	1.1 ± 0.6	1.5 ± 0.8	1.9 ± 2.4
		COG	2.1 ± 1.2	3.1 ± 1.7	3.5 ± 2.6	4.1 ± 3.1	2.2 ± 1.2	1.8 ± 1.1	3.2 ± 1.5	3.8 ± 4.2
		PR	1.9 ± 1.1	3.2 ± 2.4	2.4 ± 1.4	3.3 ± 3.2	1.7 ± 1.1	1.7 ± 0.8	2.3 ± 1.5	3.3 ± 3.9

*Note*.

*n*1, sample size of age group young‐old; *n*2, sample size of age group old‐old; RR, respiration rate (breath/min); HR, heart rate (beats/min); SCL, skin conductance level.

RR yielded a significant block effect. However, no significant time effect was obtained. HR and SCL showed a significant time effect and block effect. Regarding RR, HR, and SCL, no significant group effects and no interaction effects were observed (see Table 6). LSD post hoc tests revealed a significantly higher HR at t4 compared with t3 (*P* = 0.02, *d* = 0.62) and t2 (*P* = 0.02, *d* = 0.63). LSD values of SCL failed marginally to reach significance between t4 and t3 (*P* = 0.07, *d* = 0.49).

**Table 6:** Main effects of Time × Block × Group mixed‐factors ANOVAs for RR, HR, and SCL

Measure	Time					Block					Group				
	*df*	*F*	*P*	η²	*1‐*β	*df*	*F*	*P*	η²	*1‐*β	*df*	*F*	*P*	η²	*1‐*β
RR	3, 48	2.29	0.09	0.13	0.54	2, 32	156.27	<0.001^b^	0.91	1.00	1, 16	0.28	0.60	0.02	0.08
HR	1.92, 30.76	4.98	0.01^a^	0.24	0.76	1.32, 21.14	22.90	<0.001^b^	0.59	0.99	1, 16	0.07	0.80	0.01	0.06
SCL	1.97, 27.61	3.42	0.048^a^	0.20	0.59	2, 28	34.19	<0.001^b^	0.71	1.00	1, 14	0.35	0.56	0.03	0.09

*Note*.

a *P* < 0.05.

b *P* < 0.001.

RR, respiration rate (breath/min); HR, heart rate (beats/min); SCL, skin conductance level.

## 4 DISCUSSION

The present research examined differences in long‐term changes of cognitive performance and psychophysiological stress regulation between young‐old and old‐old successful agers. A special research design including three measurements over a period of 6 months and one measurement 6 years later was applied to account for practice effects in the cognitive tasks and habituation effects in psychophysiological responses.

At first glance, significant time‐dependent alterations occurred in young‐olds and old‐olds regarding cognitive performance and psychophysiological activity. These effects, however, should be interpreted with caution considering possible practice and habituation effects. Taking practice effects into account, general attention, multiple choice reaction performance under time pressure, and information processing appear to remain relatively stable over a period of 6 years, even in septuagenarians. There is also no evidence of an aging‐associated change in stress regulation among young‐olds and old‐olds.

### 4.1 Cognitive performance

The first three measurements conducted within a timeframe of 6 months deliver information on practice effects. Due to the fact that psychosocial characteristics^39^ and physical activity patterns remained stable during the three initial measurements, it is most likely that short‐term changes in cognitive performance are elicited to a high extent through task repetition. Thus, findings of the follow‐up test reflecting long‐term changes are interpreted based on all three initial measurements.

Prior to the cognitive measurements, motivational well‐being was recorded to control for changes that might have an impact on cognitive performance.^40^ Positive activation reflecting enthusiasm, high motivation, high energy levels, and alertness was significantly lower in the follow‐up test compared to the first three initial measurements; thus, a motivational influence on cognitive performance cannot be entirely excluded. Findings of this study, however, give no reason for this assumption, as no compelling evidence of an aging‐associated cognitive decline was observed.

General attention ability as indexed by the number of correct reactions yielded a significant long‐term improvement when compared to the first and the second measurements. The execution of the task two times prior to the third initial measurement resulted in no significant long‐term change. At this point, the question arises whether general attention ability improved or remained stable over time. A clear answer to this question cannot be given because of the uncertainty to which degree practice effects still exist in the follow‐up test. In a previous study, it was shown that practice effects persist as long as 7 years; however, with increasing age this effect decreases.^20^ Furthermore, no aging‐associated effect on the number of errors in the COG was observed. Thus, findings are interpreted in the manner of providing evidence of an unaltered general attention ability, not only in young‐olds, but also in elderly individuals in their 70s who possess numerous protective behavioral and psychological resources as reported and discussed in the article on the entire study design included in this supplement.

Performance in multiple choice reactions under time pressure demonstrated merely a significant decrease from the third measurement to the follow‐up test. According to the applied reaction time task, a previous study observed increasing practice effects of long‐term intervals between measurements (12 months) even after the second repetition of the task in athletes.^40^ Thus, it is most likely that the difference between the third measurement and the follow‐up test is a consequence of a higher practice effect in the third testing that is diminished over the long‐term period. For this reason, it is concluded that multiple choice reaction performance stayed stable over 6 years, an effect that was present in both age groups.

Information processing time appears to increase as indexed by significantly shorter processing times in the second and third measurements compared to the follow‐up test. There is the likelihood that practice effects were not present at the time of the follow‐up test, and as a result the information processing time increased. The likelihood exists that practice effects vanished in the follow‐up test, and therefore information processing time increases. Compared to the first testing, no difference in information speed was observed leading to the conclusion that information processing, if any, only declined marginally during a period of 6 years in young‐olds and old‐olds.

It is remarkable that findings revealed no deviating changes between young‐old and old‐old individuals over a time span of 6 years. Hence, the old‐old persons of this study (age at follow‐up: M ± SD = 74.8 ± 2.3 years) appear to have postponed losses in cognition similar to the young‐olds (age at follow‐up: M ± SD = 69.1 ± 1.5 years). Conversely, previous studies demonstrated significant cognitive decreases around the age of 60 years or even younger.^3^ Data of the present study give no information of the period prior to 60 years of age; consequently, it is possible that some degree of cognitive loss may have already taken place. Nevertheless, the tasks of this study differ from those commonly used in large projects on aging.^6^ Sample specifics consisting of high functioning—in terms of physical activity, and psychosocial status—elderly persons may have contributed to the postponement in cognitive decline. Thus, this result is in line with the assumption that an intellectually and socially engaged and physically active lifestyle has a positive effect on successful aging.^11^, ^15^ Multi‐ and interdisciplinary studies on aging are needed to identify patterns that support the maintenance of a high physical and mental health status aiming at providing scientific knowledge to policy makers.

### 4.2 Psychophysiological stress regulation

The applied approach of recording psychophysiological parameters prior to, during, and after the administration of cognitive tasks elicited RR, HR, and SCL increases to the stressor, and subsequent decreases in the recovery phase. Hence, both groups of elderly persons were able to show a psychophysiological pattern in terms of reactivity to and recovery from the mental stressor, indicating a physiologically unobtrusive stress regulation.^42^ There is weak evidence of aging‐related changes in cardiovascular regulation. This assumption refers to a generally higher HR at the time of the follow‐up test compared with t3. The trend of an increasing SCL in the same period partially supports HR increase. These observations are in line with previous studies showing an aging‐related increase of sympathetic activity.^25^ This obtained effect, however, might be more a result of a habituation effect to the stressors within the three initial measurements, and a possible mitigation of this effect during a period of more than 5 years, than an expression of an aging‐related change in stress regulation. It is most likely that the active lifestyle of study participants is a beneficial factor for the stability of psychophysiological stress regulation up to the age of 70 years and older. Thus, further studies using more sophisticated research designs are warranted to address the issue of alterations in psychophysiological stress regulation in successful aging, which is discussed as “a multidimensional concept that covers physical, biological and mental health, but also cognitive function, social engagement, productivity, personal control and satisfaction with life” (p. 366).^43^

### 4.3 Limitations

Unconventional cognitive tasks were used in this study. The administration of dual tasks, which were shown to be age‐sensitive,^44^ might be more useful in determining aging‐dependent changes. Thus, the findings are scarcely comparable with those of previous research on aging‐related decline. Furthermore, it cannot be excluded that commonly used tests might result in deviant findings. The sample of this study is a highly selective one and furthermore a comparison group consisting of individuals of lower physical activity is missing. Thus, conclusions should be drawn cautiously when incorporating the sample specifics, the high dropout rate, and small sample size causing low statistical power. A further weakness of the study is that nutritional behavior of the participants was not assessed, which could have influenced cognitive function^45^ and mood states.^46^ A generalization of the results is not recommendable. Finally, this research did not provide information on neurophysiological mechanisms that might explain the preservation in cognitive performance.

## 5 PERSPECTIVE

A specific sample of successful elderly agers demonstrated that aging‐associated decline in basal cognitive functioning and acute stress regulation does not unequivocally arise prior to an age of 70 years. The results underline the high significance of subjective health and psychosocial factors, as well as physical activity for maintaining cognition and the ability to cope with stressful mental situations up to advanced age. Consequently, policies are needed to establish a health‐promoting environment fostering quality of life and a lifestyle of mental and physical challenge. Furthermore, this study corroborates previous findings^21^, ^47^ on the influence of practice effects that has to be considered in longitudinal studies.
